# Supplementary material for: Instruments of Child-to-Parent Violence: Systematic Review and Meta-Analysis
Source: Healthcare (Basel). 2023 Dec 18;11(24):3192. doi: 10.3390/healthcare11243192 (PMC10743041; doi:10.3390/healthcare11243192)
Supplement: Supplementary file 1 [file healthcare-11-03192-s001.zip › healthcare-2695202-supplementary.pdf]

Supplementary Table S1:

Search phrases used for the systematic review

Database	Search terms	n
WEB OF SCIENCE	Topic=("Child-to-parent violence*" OR "child to parent violence*" OR "child-to-parent aggression*" OR "child-to-parent abuse*" OR "adolescent-to-parent violence*" OR "violent child-to-parent*" OR "adolescent violence towards parents*" OR "parent abuse*" OR "children violence towards parents*" OR "adolescent to-parent abuse*" OR "violence against parents*" OR "Children violence against parents*" OR "Adolescent violence against parents*" OR "Parent abuse offense*" OR "Child-parent violence*" OR "Child/parent violence*" OR "Child-parent aggression*" OR "youth-to-parent aggression*" OR "youth-to-parent violence*" OR "youth-to-parent abuse*" OR "youth aggression toward parents*" OR "youth violence toward parents*" OR "child-to-mother aggression*" OR "child-to-father aggression*" OR "teenage violence toward parents*" OR "adolescent-to-parent aggression*" OR "adolescent-parent abuse*" OR "adolescent aggression toward parents*" OR "adolescent violence toward parents*" OR "adolescent abuse toward parents*" OR "child-to-father violence*" OR "child-to-mother violence*" OR "child initiated family violence*" OR "adolescent-initiated parent abuse*" OR "battered parent*" OR "juvenile domestic violence*" OR "adolescent family violence*" OR "youth violence in the home*" OR "teen violence toward mothers*" OR "parents abused by children*" OR "adolescent violence in the home*" OR "parent-directed aggression*" OR "violence children against mothers*" OR "aggression toward mothers*" OR "aggression toward fathers*" OR "mother abuse*" OR "abuse toward mothers*" OR "filio parental violence*" OR "violence by children toward parents*" OR "violence by adolescents toward parents*" OR "parents abused by their children*" OR "abuse of parents by their adolescent*" OR "violence by children against parents*" OR "violence by child to parent*" OR "violence by adolescent to parent*" OR "aggression by child to parent*" OR "parents victimized by their children*" OR "parental abuse*" OR "child-to-parent violences*")	827
SCOPUS	TITLE-ABS-KEY ("Child-to-parent violence*" OR "child to parent violence*" OR "child-to-parent aggression*" OR "child-to-parent abuse*" OR "adolescent-to-parent violence*" OR "violent child-to-parent*" OR "adolescent violence towards parents*" OR "parent abuse*" OR "children violence towards parents*" OR "adolescent to-parent abuse*" OR "violence against parents*" OR "Children violence against parents*" OR "Adolescent violence against parents*" OR "Parent abuse offense*" OR "Child-parent violence*" OR "Child/parent violence*" OR "Child-parent aggression*" OR "youth-to-parent aggression*" OR "youth-to-parent violence*" OR "youth-to-parent abuse*" OR "youth aggression toward parents*" OR "youth violence toward parents*" OR "child-to-mother aggression*" OR "child-to-father aggression*" OR "teenage violence toward parents*" OR "adolescent-to-parent aggression*" OR "adolescent-parent abuse*" OR "adolescent aggression toward parents*" OR "adolescent violence toward parents*" OR "adolescent abuse toward parents*" OR "child-to-father violence*" OR "child-to-mother violence*" OR "child initiated family violence*" OR "adolescent-initiated parent abuse*" OR "battered parent*" OR "juvenile domestic violence*" OR "adolescent family	622

	violence*" OR "youth violence in the home*" OR "teen violence toward mothers*" OR "parents abused by children*" OR "adolescent violence in the home*" OR "parent-directed aggression*" OR "violence children against mothers*" OR "aggression toward mothers*" OR "aggression toward fathers*" OR "mother abuse*" OR "abuse toward mothers*" OR "filio parental violence*" OR "violence by children toward parents*" OR "violence by adolescents toward parents*" OR "parents abused by their children*" OR "abuse of parents by their adolescent*" OR "violence by children against parents*" OR "violence by child to parent*" OR "violence by adolescent to parent*" OR "aggression by child to parent*" OR "parents victimized by their children*" OR "parental abuse*" OR "child-to-parent violences*")	
PSYCINFO	All fields=("Child-to-parent violence*" OR "child to parent violence*" OR "child-to-parent aggression*" OR "child-to-parent abuse*" OR "adolescent-to-parent violence*" OR "violent child-to-parent*" OR "adolescent violence towards parents*" OR "parent abuse*" OR "children violence towards parents*" OR "adolescent to-parent abuse*" OR "violence against parents*" OR "Children violence against parents*" OR "Adolescent violence against parents*" OR "Parent abuse offense*" OR "Child-parent violence*" OR "Child/parent violence*" OR "Child-parent aggression*" OR "youth-to-parent aggression*" OR "youth-to-parent violence*" OR "youth-to-parent abuse*" OR "youth aggression toward parents*" OR "youth violence toward parents*" OR "child-to-mother aggression*" OR "child-to-father aggression*" OR "teenage violence toward parents*" OR "adolescent-to-parent aggression*" OR "adolescent-parent abuse*" OR "adolescent aggression toward parents*" OR "adolescent violence toward parents*" OR "adolescent abuse toward parents*" OR "child-to-father violence*" OR "child-to-mother violence*" OR "child initiated family violence*" OR "adolescent-initiated parent abuse*" OR "battered parent*" OR "juvenile domestic violence*" OR "adolescent family violence*" OR "youth violence in the home*" OR "teen violence toward mothers*" OR "parents abused by children*" OR "adolescent violence in the home*" OR "parent-directed aggression*" OR "violence children against mothers*" OR "aggression toward mothers*" OR "aggression toward fathers*" OR "mother abuse*" OR "abuse toward mothers*" OR "filio parental violence*" OR "violence by children toward parents*" OR "violence by adolescents toward parents*" OR "parents abused by their children*" OR "abuse of parents by their adolescent*" OR "violence by children against parents*" OR "violence by child to parent*" OR "violence by adolescent to parent*" OR "aggression by child to parent*" OR "parents victimized by their children*" OR "parental abuse*" OR "child-to-parent violences*")	520
PSYCARTICLES		233
TOTAL		2202

Supplementary Figure S1. Cronbach's Aplha Global sensitivity analysis

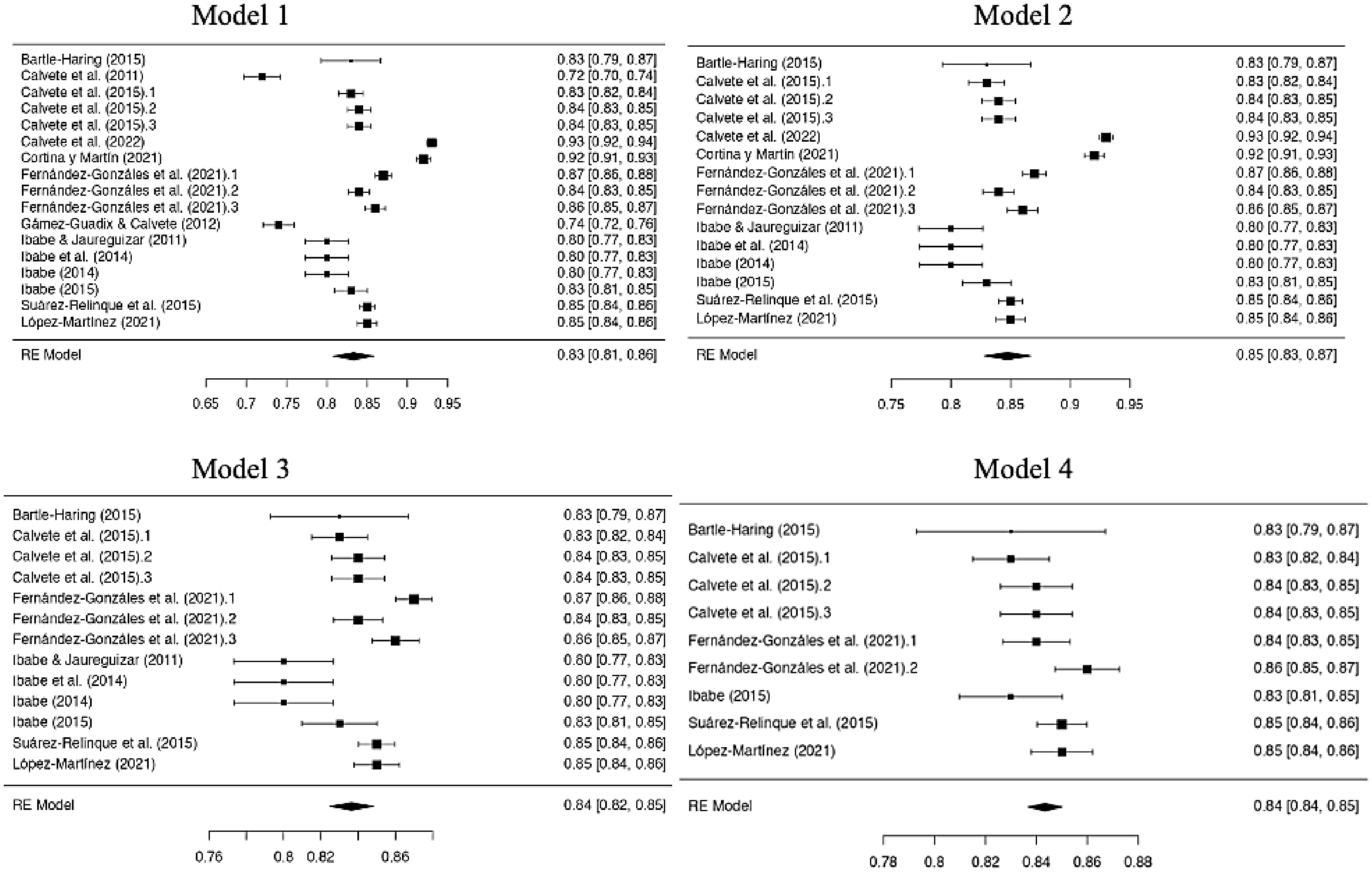

In Model 1, the Egger coefficient was -3.261 and I2 was 98.45%. This is the initial model. In the second model, studies with an alpha less than .80 were removed. Minor differences were found in the coefficients, with Egger = -4.226 and I2 = 97.28%. In the third model, studies with an alpha greater than .90 were eliminated, resulting in coefficients of Egger = -5.045 and I2 = 86.89%. In Model 4, studies with alpha values less than .83 and greater than .85 were removed. This yielded an Egger = -1.879 and I2 = 49.41%. Thus, outliers appear to influence the initial model. However, these results should be cautiously interpreted. This will be a priority objective for future studies the following year.
